# Variant Rabbit Hemorrhagic Disease Virus in Young Rabbits, Spain

**DOI:** 10.3201/eid1812.120341

**Published:** 2012-12

**Authors:** Kevin P. Dalton, Inés Nicieza, Ana Balseiro, María A. Muguerza, Joan M. Rosell, Rosa Casais, Ángel L. Álvarez, Francisco Parra

**Affiliations:** Universidad de Oviedo Instituto Universitario de Biotecnología de Asturias, Oviedo, Spain (K.P. Dalton, I. Nicieza, A.L. Álvarez, F. Parra);; Centro de Biotecnología Animal (SERIDA), Gijón, Spain (A. Balseiro, R. Casais);; INTIA División ITG, Navarra, Spain (M.A. Muguerza); and Cunivet Service, Tarragona, Spain (J.M. Rosell)

**Keywords:** RHDV, variant, rabbits, rabbit hemorrhagic disease virus, fatal, viruses, Iberian Peninsula, Spain, rabbit hemorrhagic disease, RHD, RHDV-N11

## Abstract

Outbreaks of rabbit hemorrhagic disease have occurred recently in young rabbits on farms on the Iberian Peninsula where rabbits were previously vaccinated. Investigation identified a rabbit hemorrhagic disease virus variant genetically related to apathogenic rabbit caliciviruses. Improved antivirus strategies are needed to slow the spread of this pathogen.

Rabbit hemorrhagic disease (RHD) is rapidly fatal, with mortality rates of 70%–100% in adult rabbits (*1*); young rabbits (kits) are unaffected or subclinically infected (*1*,*2*). This difference in disease susceptibility is poorly understood, but it may be due to changes in tissue-specific receptors that occur as young rabbits develop to adulthood (*3*).

RHD is caused by *Rabbit hemorrhagic disease virus* (RHDV; genus *Lagovirus*, family *Caliciviridae*) (*4*), a virus with a positive-sense, single-stranded RNA genome of 7.4 kb. The single serotype of RHDV is divided into 2 subtypes, classic RHDV and RHDVa. Effective inactivated vaccines prepared from liver extracts of rabbits experimentally infected with classic RHDV strains are used as a prophylactic and postoutbreak strategy to combat disease (*1*).

RHDV is not cultivatable in cell culture; therefore, detection of virus genome, virions, and anti-RHDV antibodies and experimental infection of rabbits are required for diagnosis and virus characterization (*1*). Sequence regions of the major capsid protein viral protein (VP) 1 are used to type and classify strains.

The identification of rabbit caliciviruses (RCVs) (*5*,*6*), nonpathogenic viruses antigenetically similar to RHDV, and recent descriptions of a pathogenic RCV (*7*), an RHDV variant grouping with RCV viruses in phylogenetic analysis (*8*), and nonpathogenic RHDV (*9*) raise questions about the origins, classification, and nomenclature of these viruses. On the Iberian Peninsula, RHDVa or pathogenic or nonpathogenic RCV isolates had not been reported (*10*). We report the results of an investigation of outbreaks of RHD among young rabbits on farms on the Iberian Peninsula where rabbits were previously vaccinated for RHDV.

## The Study

During September 2011–February 2012, our laboratory received liver samples from 9 rabbitries from 3 areas of northeastern Spain where acute outbreaks of RHD were occurring in adult rabbits and kits. We analyzed 35 tissue samples from 20 kits (age 14–35 days), 9 growers (age 36–57 days), and 6 adults. Macroscopic lesions in infected kits were consistent with RHDV infection usually observed only in adult rabbits (*4*). The lesions in young rabbits consisted of hemorrhages in heart, trachea, thymus, lungs, liver, kidneys, and gut; jaundice was also seen. Mortality rates of up to 20% and 50% in adult and young rabbits, respectively, were observed. Infected samples came from vaccinated (n = 23) and unvaccinated young and adult rabbits.

Reverse transcription PCR was performed by using RNA extracted from 20 mg of liver samples using the mini RNAeasy RNA extraction kit (QIAGEN Iberia, Madrid, Spain), Superscript III reverse transcription (Invitrogen Corp., Carlsbad, CA, USA), LA-Taq polymerase (Takara Bio, Otsu, Japan), and forward and reverse primers annealing at nt 6056–6075 and 6775–6794, respectively (positions refer to the genomic sequence of RHDV Ast/89; GenBank accession no. Z49271). A band of the expected size (738 bp) was purified after gel electrophoresis and sequenced; this isolate was named RHDV-N11 and deposited into GenBank (accession no. JX133161). The sequenced region consisted of nt 6108–6716, corresponding to domains CDE and partial B and F domains of the VP1 capsid protein (*11*). Sequence analysis showed that samples from each farm contained the same virus (96.6% identity) and that the virus detected had 81% identity with both RHDV and RHDVa sequences (data not shown).

**Figure 1:**
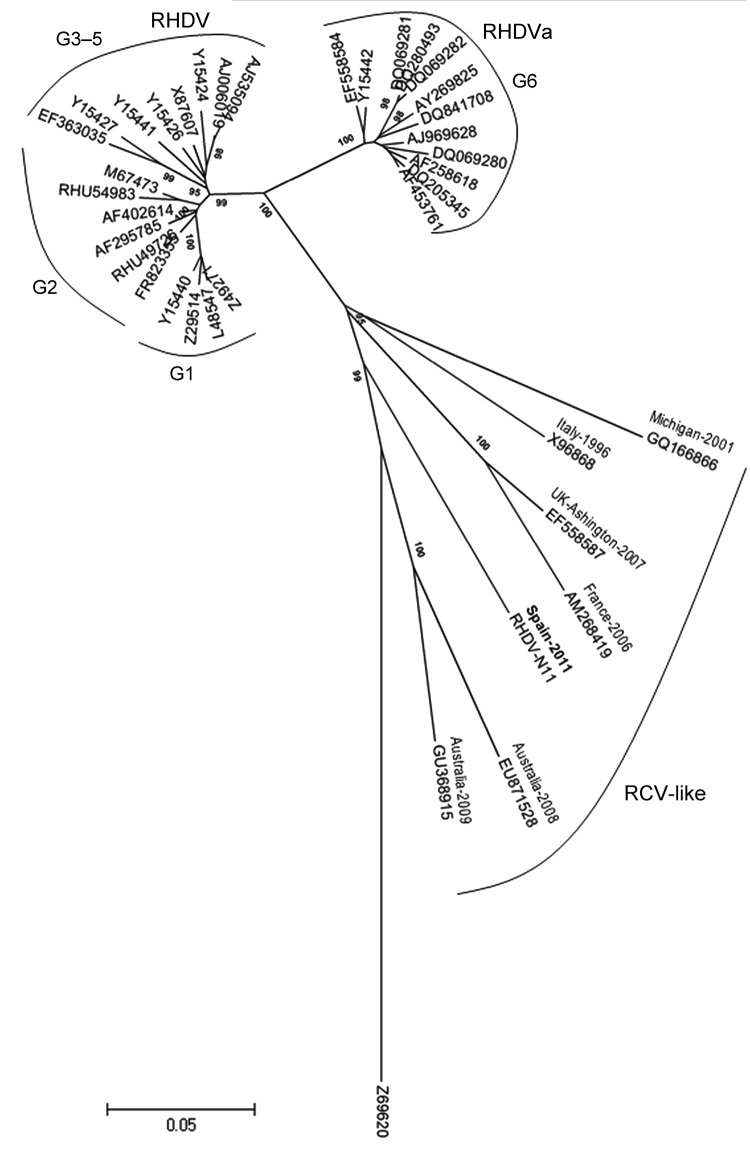
Evolutionary relationships of rabbit hemorrhagic disease virus (RHDV) and related viruses. A total of 38 nt sequences were analyzed: the isolate from this study, designated RHDV-N11 (GenBank accession no. JX133161); 18 classical RHDV and 12 RHDVa isolates; 6 rabbit calicivirus (RCV)–like isolates; and European brown hare syndrome virus (GenBank accession no. Z69620) as an outlier. Evolutionary history was inferred by using the neighbor-joining method; optimal tree with the sum of branch length = 1.33075964 is shown. The tree is drawn to scale, with branch lengths in the same units as those of the evolutionary distances used to infer the phylogenetic tree. Evolutionary distances were computed by using the p-distance method (*14*) and are in the units of the number of base differences per site. Codon positions included were 1st + 2nd + 3rd + Noncoding. All ambiguous positions were removed for each sequence pair; the final dataset consisted of 646 positions. Evolutionary analyses were conducted in MEGA5 (*15*). RHDV genetic groups are indicated. GenBank accession numbers of the sequences were: RCV-like strains: EU871528, GU368915, X96868, EF558587, AM268419, GQ166866; RHDV strains; AJ006019, AJ535094, Y15424, Y15426, Y15441, EF363035, Y15427, AF402614, RHU54983, AF295785, Z49271, L48547, Y15440, RHU49726, Z29514, M67473, X87607, FR823355; RHDVa strains: DQ069280, DQ280493, DQ069282, AY269825, DQ841708, DQ205345, AF258618, DQ069281, AJ969628, AF453761, EF558584, Y15442.

Multiple sequence alignment and phylogenetic analysis were performed by using the RHDV-N11 VP1 sequence and 37 other sequences (18 classic RHDV, 12 RHDVa, and 6 RCV-like, with European brown hare syndrome virus as an outlier). The RHDV-N11 sequence was shown to form a branch falling between RCV and RCV-A1, separated from RHDV and RHDVa (Figure 1). We suggest the term RHDVb for this new isolate type.

To confirm the presence of virions in the infected livers, liver homogenates (10% in sterile phosphate-buffered saline) were clarified by low-speed centrifugation followed by ultracentrifugation through a 30% sucrose cushion. Pellets were suspended in phosphate-buffered saline for further study. A major band of ≈60 kDa was observed by sodium dodecyl sulfate–polyacrylamide gel electrophoresis and detected by Western blot by using a rabbit polyclonal antibody against RHDV Ast/89 (*4*). Dot-blot analyses using monoclonal antibodies 1H8 and 6G2 (*12*) revealed that, although RHDV Ast/89 reacted with both monoclonal antibodies, the new RHDV-N11 isolate reacted with 6G2 only (data not shown). This type of reactivity (negative 1H8, positive 6G2) was found (L. Capucci, pers. comm.) for a recent French variant (*8*).

Agglutination studies showed that RHDV Ast/89 agglutinated human blood of all group types (O, A, B, and AB), as described for RHDV genetic group 1 members (*13*). RHDV-N11 showed no agglutination of blood groups O or A, but did agglutinate blood groups B and AB; this pattern is similar that of G4 and G6 groups (*13*) (Table 1).

**Table 1:** Agglutination titers for RHDV (Ast/89) and RHDV-N11, by human blood type, Spain

Virus	Human blood type†			
	O	A	B	AB
RHDV (Ast/89)	32,768	16,384	131,072	131,072
RHDV-N11	<2	<2	2,048	2,048

*RHDV, rabbit hemorrhagic disease virus. †0.5% in phosphate-buffered saline at 4°C.

To prove that the virus isolated was the etiologic agent of RHDV in these animals, we conducted a small-scale experiment. Six New Zealand white rabbits, 3 kits (30 days of age) and 3 adult rabbits were experimentally infected in a biosecurity level 2 laboratory with 15,000 hemagglutination units of purified RHDV-N11 virions (using B-type human erythrocytes). One additional adult and kit were used as uninoculated controls. Two routes of infection were used: 2 adult and 2 kits were infected subcutaneously, and 1 adult and 1 kit were infected intranasally. Forty-eight hours postinfection, 2 rabbits (1 adult and 1 kit) died. Postmortem analysis revealed discoloration of the liver and extensive hemorrhaging in the lungs. Ninety-six hours postinfection, the kit control died, also showing macroscopic lesions consistent with RHDV.

**Figure 2:**
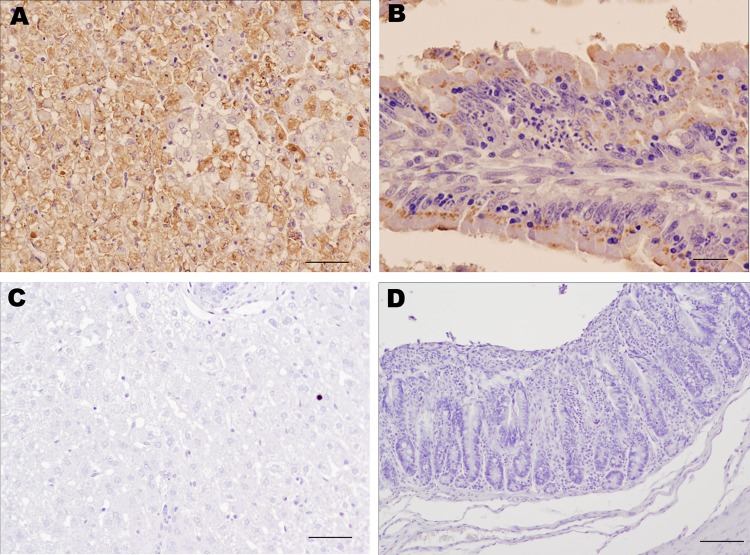
Results of immunohistochemical staining using monoclonal antibody 6G2 and the ABC complex technique of liver and intestine samples from young rabbits infected with rabbit hemorrhagic disease virus (RHDV) isolate RHDV-N11 and control rabbits. A) Liver of RHDV-N11–infected rabbit. Hepatocytes show intense 6G2-specific immunolabeling. Scale bar = 50 µm. B) Intestinal villi in small intestine of RHDV-N11–infected rabbit. Areas of focal necrosis and epithelial cells show strong immunolabeling. Scale bar = 20 µm. C) Liver of control rabbit. Hepatocytes do not show positive immunolabeling. Scale bar = 50 µm. D) Epithelial cells of intestinal villi of control rabbit do not show positive results on staining. Scale bar = 100 µm.

Immunohistochemical staining of tissues using the anti-RHDV mouse monoclonal 6G2 (1:700) and the Avidin-Biotin complex (Vectastain ABC Kit; Vector Laboratories Ltd., Peterborough, UK) confirmed the presence of RHDV-N11 VP1 in the liver, heart, kidney, spleen, lung, and intestine of infected rabbits. Data for RHDV-N11 antigen detection in the experimentally infected rabbits are shown in Table 2. Immunohistochemical analyses of tissues from the subcutaneously inoculated kit that died 48 h postinfection show hepatocytes with intense RHDV-specific immunolabeling (Figure 2, panel A). Areas of focal necrosis and epithelial cells showing strong immunolabeling were also observed in the intestinal villi in the small intestine (Figure 2, panel B).

**Table 2:** Immunohistochemical detection of RHDV-N11 in tissues from experimentally infected and control rabbits by using monoclonal antibody 6G2*

Rabbit no. and age	Infection method	Tissue					
		Liver	Lung	Kidney	Spleen	Intestine	Heart
1. Adult (control)	SC	–	–	–	–	+/–	–
2. Young (control)†	SC	+	++	++	+	++	+/–
3. Young	SC	–	–	–	–	–	–
4. Adult‡	SC	+	+	++	+++	+	+/–
5. Young‡	SC	+++	+	++	+++	++	+/–
6. Adult	SC	–	–	–	–	–	–
7. Adult	IN	–	–	–	–	+	–
8. Young	IN	–	–	–	–	–	–

*Young rabbits were 30 d of age on the day of experimental infection. Control rabbits (1 and 2) were each injected with 500 µL sterile PBS. RHDV, rabbit hemorrhagic disease virus; PBS, phosphate-buffered saline; SC, subcutaneous injection; IN, intranasal administration; –, not detected; +, ++, +++, increasing levels of antigen. †Died 96 h after experimental infection of animals 3–8. ‡Died 48 h after experimental infection.

RNA extracted from the livers of the rabbits that died after experimental RHDV infection was analyzed by RT-PCR. These samples were confirmed to contain virus sequences corresponding to those of the new variant RHDV.

## Conclusions

A variant of RHDV has been detected on the Iberian Peninsula in Spain and found to be responsible for causing disease and death in kits <30 days of age, even though rabbits of this age were not known to be susceptible to illness caused by RHDV infection. The RHDV-N11 variant differs antigenically from classic RHDV, as determined by dot-blot analyses using monoclonal antibodies and hemagglutination analysis. Phylogenetic analysis suggests that this virus isolate is genetically distant from RHDV and RHDVa and is more closely related to the so-called RCV apathogenic viruses. On the basis of these data, we suggest the use of the term RHDVb for this variant.

The occurrences of these outbreaks on farms where rabbits were previously vaccinated against RHDV raises serious concerns about the efficacy of the vaccines. Mutations of critical amino acids that result in antigenic differences, as suggested by the different monoclonal antibody recognition patterns, could explain why the immune response in vaccinated rabbits did not protect against RHDV-N11. Changes in receptor usage, as suggested by the modified agglutination patterns, may explain the different pathogenicity of this variant RHDV. These findings suggest the need to improve prophylactic reagents and reinforce hygienic measures to avoid the spread of this reemerging pathogen.
